# A Two-Step Femtosecond Laser-Based Deposition of Robust Corrosion-Resistant Molybdenum Oxide Coating

**DOI:** 10.3390/ma16030909

**Published:** 2023-01-18

**Authors:** Asghar Ali, Piotr Piatkowski, Tahir Nawaz, Shahbaz Ahmad, Taleb Ibrahim, Mustafa Khamis, Ali S. Alnaser

**Affiliations:** 1 Department of Physics, American University of Sharjah, Sharjah 26666, United Arab Emirates; 2 Materials Science and Engineering Program, College of Arts and Sciences, American University of Sharjah, Sharjah 26666, United Arab Emirates; 3 Department of Chemical Engineering, College of Engineering, American University of Sharjah, Sharjah 26666, United Arab Emirates; 4 Department of Biology, Chemistry, and Environmental Sciences, College of Arts and Sciences, American University of Sharjah, Sharjah 26666, United Arab Emirates

**Keywords:** femtosecond laser, structuring, coating, corrosion

## Abstract

A two-step femtosecond-pulsed laser deposition (fs-PLD) process is reported for the rapid development of uniform, poreless, crack-free, and well-adhering amorphous coatings of source materials with a high melting point. The first step comprises a high-rate raw deposition of the source material via fs-PLD, followed by a second step of scanning the raw sample with fs laser pulses of optimized fluence and scan parameters. The technique is applied to develop substoichiometric molybdenum oxide (MoO_x_, x < 3) coatings on mild steel. The thickness of the layer was ~4.25 μm with roughness around 0.27 μm. Comprehensive surface characterization reveals highly uniform and relatively moderate roughness coatings, implying the potential of these films as robust corrosion-resistant coats. Corrosion measurements in an aqueous NaCl environment revealed that the coated mild steel samples possess an average corrosion inhibition efficiency of around 95% relative to polished mild steel.

## 1. Introduction

Utilization of femtosecond lasers in material processing allows for rapid energy transfer into the material on femtosecond and picosecond time scales, which is extremely fast compared to the plasma expansion time that occurs in the nanosecond regime. This ultrafast energy transfer process leads to localized heating of the target material with a rapid rise in temperature and minimal dissipation or damage to the surrounding area [1]. The ultrashort pulse duration and high peak laser intensity combined with the rich manipulation parameters, such as the laser power and fluence, the spot size and focus, state of polarization, repetition rate, pulse width, pulse energy, scan speed, line spacing, etc., allow for the extensive exploitation of femtosecond lasers in fundamental as well as applied fields of research [2]. Femtosecond lasers are often employed in photo-emission spectroscopy [3], pumping optical parametric amplifiers [4], high harmonics generation [5], pump-probe experiments [6], terahertz generation [7], life and health sciences [8], and materials processing [9].

Materials processing using femtosecond lasers was first demonstrated in 1987 by Küper and Stuke [10], and Srinivasan et al. [11]. At present, femtosecond lasers are used for micro and nanoprocessing of materials for optics [12], electronics [13], optoelectronics [14], photonics [15], photovoltaics [16], photolithography [17], microchanneling [18], optofluidics [19], optomechanics [20], additive manufacturing [21], surface micro- and nanostructuring [22], glass welding [23], corrosion inhibition [24], and tissue engineering [25].

There have been extensive studies in the literature on femtosecond surface ablation, texturing, and surface treatment. Zhao et al. [26] investigated the surface reflectivity in correlation with the ablation mass removal of a polished poly-Mo target with fs laser pulses. They demonstrated that the surface reflectivity suffers a profound decline for the first few laser pulses applied at different fluence values. Kotsedi et al. [27] developed Mo thin films on corning glass, employing electron beam evaporation, and studied the fs laser surface structuring of these films with a manifestation of the change in color and selective photo-absorbing nature with an fs laser. In a separate study, Kotsedi et al. [28] deposited Mo thin films on glass with electron beam evaporation and subjected them to an fs laser in the air to develop molybdenum oxide nanorods on the surface. Cano-Lara et al. [29] sputter deposited Mo thin films on fused silica and irradiated the sample with low energy (nJ) and high repetition rate (MHz) fs laser pulses. They demonstrated that scanning with such pulses induces different crystalline phases along the surface and its vicinity. A comprehensive review on fs laser surface structuring is given by A.Y. Vorobyev and C. Guo [30].

There are several challenging areas where femtosecond lasers may be successfully employed, including perforation, drilling, and thin film-coating deposition [31,32]. Femtosecond lasers are generally the preferred choice for structuring surfaces [2]; however, thin film coatings with femtosecond laser-based deposition (fs-PLD) are a relatively new concept and needs to be exploited further.

Conventional thin film deposition techniques suffer from many limitations, and more advanced techniques are required for further advances in thin film deposition technology. For example, resistive thermal evaporators are not designed to evaporate refractory metals or high-temperature ceramics; and achieving thicker films with good adhesion remains challenging [33]. Sputter systems are generally meant for thin film deposition; however, when high powers are used to achieve reasonable deposition rates, melting of the surrounding holder accessories or cracking in the target material may occur. Moreover, targets of specified dimensions are required, and target consumption is uneven. Depending on the nature of the sample, switching between DC and RF sputtering could be necessary. Likewise, using high power can break the chemical bonds between the source atoms during sputtering, and thus stoichiometry becomes a concern in these cases. Working gas pressure also disturbs the deposition rate and may impact film quality [34]. One may opt for electron beam evaporation for thicker films since it supports higher deposition rates. However, the nature of the sample, its wettability, selecting the proper crucible, charge build-up, beam diversion, contamination of the chamber, risk of short-circuiting, especially when trying higher deposition rates, constant surveillance, and non-uniform source consumption, especially in the case of ceramics, are some of the major shortcomings [34]. For thicker coating deposition, plasma spray is one of the most versatile among the different thermal spray deposition techniques. However, bond coat strength and adhesion, porosity, voids, structure and composition, and unmelted particles still raise major concerns. Moreover, other impeding issues are damage to the substrate, process optimization, and materials wastage [35].

Compared to the many conventional and contemporary deposition techniques, a femtosecond-pulsed laser-based deposition (fs-PLD) technique can potentially deposit coatings of very high melting point solids, powders, or pellets with a high deposition rate. However, the limited literature available on the subject suggests that developing quality films has never been easy with fs-PLD and that some serious concerns need to be addressed [32,36,37,38,39]. The most common issue is the micro and nano-sized source particles [32,38,39] that exist in the fs-PLD deposited thin films, which could severely degrade the coating adhesion and uniformity. To improve adhesion, substrate heating can be carried out but, in many cases, at the cost of irreversible phase change and crystallization [38]. Film uniformity and smoothness generally cannot be fully addressed even after substrate heating. Reducing laser fluence may help reduce the particle size [32] but cannot satisfactorily address the adhesion and uniformity issues. Substrate roughening is another option to improve coating adhesion marginally, but it cannot help the ablated powder stick to the substrate [40]. The details of coating adhesion and thickness requirements for different applications have been detailed elsewhere [41].

In this work, we exploit the advantages of femtosecond laser in developing uniform, poreless, crack-free films of refractory metals such as molybdenum (Mo). We devise a two-step fs-PLD comprising rapidly depositing a raw coating on mild steel, followed by a high-speed point-by-point scan of the sample with low-power femtosecond laser pulses in the air. Considering the technological importance of Mo and its compounds [42], we demonstrate the development of well-adhering amorphous Mo oxide coatings by employing the two-step fs-PLD process, with fs laser as the sole processing tool. Coated mild steel (MS) was subjected to corrosion studies in aqueous sodium chloride (NaCl) environment to assess the quality and corrosion inhibition performance of the coating.

## 2. Experimental

The substrates were discs of mild steel of diameter~25 mm and thickness ~1.3 mm. The mild steel was ground with abrasive papers of different grit sizes, followed by polishing with a cloth and diamond paste. The source material was an Mo foil (99.9%, Sigma-Aldrich, Steinheim, Germany).

An active fiber systems’ Ytterbium-300 laser with 300 W total average power output was employed in the current study. The system, with an emission wavelength (λ) centered at 1030 nm, was operated at different values of laser power, duration, and repetition rate.

**Figure 1:**
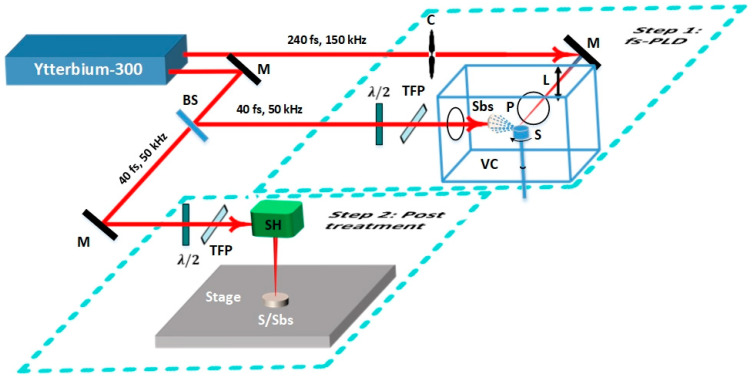
Schematic diagram of the experimental setup: Step 1—femtosecond pulsed laser deposition of the source material, Step 2—post treatment of the sample in step 1 using scan head, Ytterbium-300—300 W ytterbium laser with 240 fs, 150 kHz and 40 fs, 50 kHz laser beams, C—Optical chopper, M—dielectric mirror, L—objective lens, VC—vacuum chamber, S—source material wrapped around a rotating cylindrical holder, P—plasma plume, Sbs—substrate, BS—beam splitter, λ/2—half wave plate, TFP—thin film polarizer, SH—scan head, S/Sbs—source material deposited over the substrate via fs-PLD, Stage—*z*-axis translation stage.

The coating process was performed in two steps (see Figure 1). The fs-PLD step was performed in a rough vacuum environment. The working pressure was $3.22\times 10^{-1}$ mbar or lower. One set of experiments was carried out with the substrate heated up to 200 °C and another without substrate heating. The mild steel substrates were heated with an unfocused 40 fs pulses of beam diameter 10 mm. Gaussian laser pulses of 240 fs pulse duration, 150 kHz repetition rate, and 3 W power were employed to deposit Mo. The laser beam was focused onto a spot size ($d_{spot}$) of 70 μm to achieve 0.5 J/cm^2^ fluence on a rotating cylindrical disc with several layers of Mo foil wrapped around it. The deposition process was carried out at a rate of ~3.3 µm/min for 4 min. We name the sample prepared in this step as the as-deposited Mo/MS.

In the next step, the as-deposited Mo/MS sample was treated in air with 1.5 W, followed by 0.2 W laser power. The pulse duration was 40 fs and repetition rate was 50 kHz. For $d_{spot}$ of 50 μm, the corresponding fluence values were 1.5 and 0.2 J/cm^2^, respectively. A scan head was employed to completely scan the sample surface. A parallel line scan of scan speed ($v_{\mathrm{scan}}$) 3325 mm/s and line spacing (∆d) 10 μm (or 0.01 mm) was performed. The sample obtained after 1.5 J/cm^2^ treatment is named Mo-1.5/MS, whereas the sample obtained after 0.2 J/cm^2^ treatment of Mo-1.5/MS is termed as MoO_x_/MS.

For the purpose of structural comparison, polished MS without any coating was also laser-scanned with 1.5 J/cm^2^ followed by scanning with 0.2 J/cm^2^. The same pulse duration of 40 fs and repetition rate of 50 kHz were maintained.

The morphological and elemental studies were carried out with a TESCAN VEGA 3 LMU scanning electron microscope and an INCAx-act EDS detector from Oxford Instruments (Abingdon, UK). The 2D fast Fourier transforms (FFTs) were calculated from SEM micrographs with VegaTC 4.1.20.3 SEM software. The information about the chemical nature of the sample’s surface was acquired with a Renishaw inVia confocal Raman microscope (Wotton-under-Edge, UK) fitted with a 100× focusing lens. The excitation wavelength was 514 nm, and the power was 1.5 mW. For detailed crystallographic information and phase confirmation, X-ray diffraction analysis was conducted with an X’Pert^3^ Powder diffractometer from Panalytical (Malvern, UK) within a 2θ in the 10°−90° range. Topography and roughness measurements were acquired with hpAFM atomic force microscope from NANOMAGNETICS Instruments (Ankara, Turkey). The electrochemical measurements were performed with a CHI 660E electrochemical workstation of CH Instruments, Inc. (Austin, TX, USA). The open circuit potential ($E_{OC}$) was let to stabilize in each case. Although the stability time was variable for each type of sample, before taking any measurements, it was ensured that there was a negligible change in $E_{OC}$ with time. The scan rate was set to 0.001 V/s in the auto-sense mode. The voltage window was shifted relative to the $E_{OC}$ for each sample.

## 3. Results and Discussion

Figure S1 presents camera images of the Mo/MS produced by the raw fs-PLD process (Figure S1a), the Mo-1.5/MS obtained after laser scanning of Mo/MS at 1.5 J/cm^2^ (Figure S1a), and MoO_x_/MS produced after laser-scanning the Mo-1.5/MS (Figure S1c) with 0.2 J/cm^2^. From the physical appearance, the Mo/MS is the darkest, hinting at the presence of rough light-scattering features on the surface responsible for reduced specular reflections [43]. Similarly, the Mo coat depicts a very low packing density and reduced adhesion with the MS substrate. The film can easily be washed off. With substrate heating, film adhesion improves marginally, but the structural non-uniformity still exists.

To improve the adhesion and uniformity of the Mo/MS coating, the constituent submicron particles in the Mo layer need to be fused together. Melting and resolidification of the Mo layer can mitigate the particles as well as improve adhesion. However, heating Mo to its melting point (i.e., 2623 °C) without damaging the substrate MS (with melting point = 1350–1530 °C) is impossible with conventional resistive heating techniques. In addition to Fe melting and diffusion into Mo, coating delamination and cracks formation at high temperatures is another concern. Therefore, selective heating of the top Mo layer to melt and oxidize Mo without damaging the substrate is inevitable. Femtosecond laser can impart very high peak power pulses (up to 7 GW) for very short durations and can instantly heat up Mo. Anticipating improvement in the quality of the top Mo layer, we proceeded to laser-scan Mo/MS.

After laser scanning Mo/MS at 1.5 J/cm^2^, the top Mo coat transformed into a slightly reflecting film. This demonstrates that the film has become much smoother and uniform compared to the as-deposited Mo/MS. Subsequent laser scanning at 0.2 J/cm^2^ transformed the topcoat into an almost transparent coating with the substrate visible through it. This change in color and transparency suggests that laser-induced physical and chemical changes have taken place on the sample surface.

**Figure 2:**
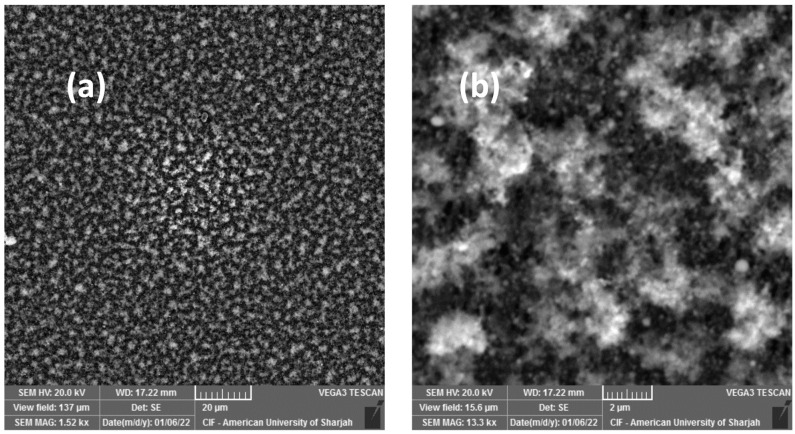
SEM micrographs of the as-deposited Mo thin film over mild steel (Mo/MS) at view fields of (**a**) 137 μm and (**b**) 15.6 μm.

SEM analysis was carried out to explore the associated morphological transformations. Figure 2 reveals that Mo/MS is actually composed of submicron particles ejected from the Mo source as ablation products. Since fs pulses have very high peak powers, such submicron ablation debris are expected [44], thus rendering very high deposition rates

**Figure 3:**
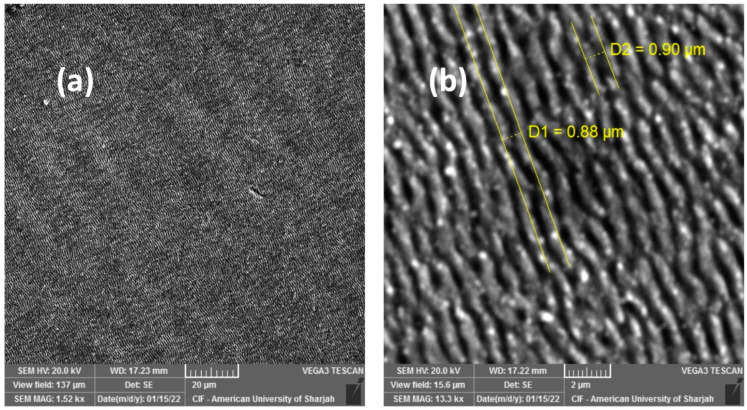
SEM micrographs at (**a**) 1.5 kx and (**b**) 13.3 kx magnifications of Mo-1.5/MS obtained after laser-scanning the as-deposited Mo/MS with 40 fs pulses of 1.5 J/cm^2^ fluence and 50 kHz repetition rate.

The surface morphology of the Mo-1.5/MS is quite different from that of Mo/MS. After the laser scan, the ablation debris are removed altogether, instead laser-induced periodic surface structures (LIPSS) with a period of around 0.9 μm occupy the surface (Figure 3). Similarly, there are no deep ablation craters suggesting minimal ablation to have taken place.

**Figure 4:**
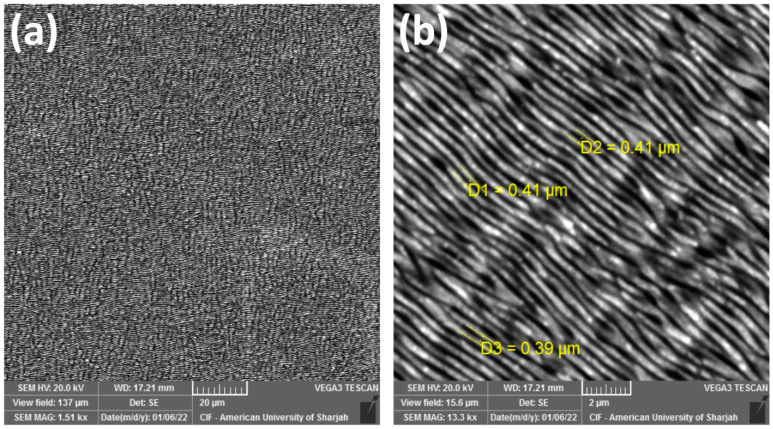
SEM micrographs at (**a**) 1.5 kx and (**b**) 13.3 kx magnifications of MoO_x_/MS obtained after laser-scanning Mo-1.5/MS with 40 fs pulses of 0.2 J/cm^2^ fluence and 50 kHz repetition rate.

Substoichiometric MoO_x_ oxidizes at temperatures as low as 300 °C [45]. Similarly, Cuando-Espitia et al. [46] demonstrated that Mo thin films oxidize with fs laser treatment in air and at different oxygen partial pressures. To oxidize Mo-1.5/MS, the sample was laser-scanned eight times in ambient air with 0.2 J/cm^2^ pulses. As shown in Figure 4, the coating is uniform throughout, crack-free, and pore-free. Moreover, more uniform and finer LIPSS of 0.4 μm periods covering the whole surface are observable. The scan with 1.5 J/cm^2^ was meant to melt the ablation debris so that they take the form of a uniform coat that strongly adheres to the MS substrate. The LIPSS that appeared due to the 1.5 J/cm^2^ treatment might have influenced the orientation and/or morphology of the subsequently appearing LIPSS due to the 0.2 J/cm^2^ treatment [47,48].

**Figure 5:**
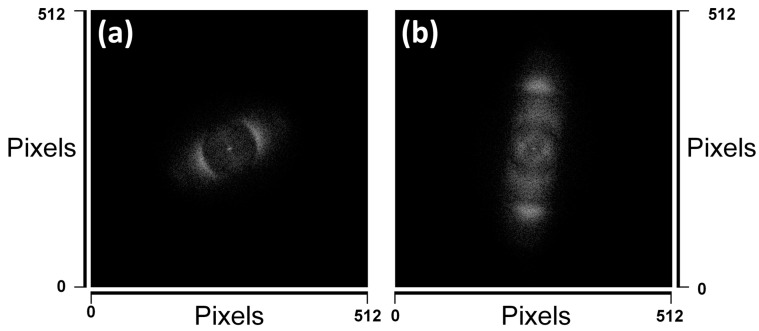
2D Fourier transform of (**a**) Mo-1.5/MS and (**b**) MoOx/MS micrographs shown in Figure S4. The view field in either case is 48.1 μm.

To analyze the periodicity and estimate the spatial period, 2D fast Fourier transforms (FFTs) of the SEM micrographs (Figure S4) of Mo-1.5/MS and MoO_x_/MS were acquired. Figure 5 details the corresponding FFTs. The period of the LIPSS observed on the Mo-1.5/MS is 0.86 μm. Since its more than half of the central laser wavelength, i.e., 1030 nm, we can categorize these as low spatial frequency LIPSS (LSFL) [49]. There are no other prominent periodic or quasi-periodic structures present on Mo-1.5/MS surface. On the other hand, the MoO_x_/MS depicts three different spatial periods correlatable with three distinct surface features. The most noticeable are the 0.41 μm LIPSS. Since the spatial period is less than half of the laser wavelength, we can categorize these as high spatial frequency LIPSS (HSFL) [49]. The other type of periodic surface features has an average spatial period close to 0.73 μm. These features are the remains of the LSFL which appeared on Mo/MS after 1.5 J/cm^2^ treatment. Their presence is somewhat overshadowed by the presence of HSFL in the corresponding SEM micrograph (Figure 4b) because multiple scans with 0.2 J/cm^2^ washed off the underlying LSFL almost completely. Similarly, the coinciding alignment directions of LSFL and HSFL as suggested in the FFT makes LSFL even less obvious in the SEM micrograph. The last among these features depicts a spatial period of around 1.7 μm. These features are identified in the SEM micrographs as the bigger wavy features aligned perpendicular to both LSFL and HSFL (Figure 4b) and Figure S4b). Since well-developed quasi-periodic grooves have a spatial period of 2–4 times that of the laser wavelength [50], and usually require multiple number of pulses (multiple scans in this case) [51,52], we believe that these features are grooves rudiments. It is known that reducing pulse fluence [53,54] and/or increasing the number of pulses [55] can result in the emergence of HSFL, therefore, HSFL found on MoO_x_/MS may either be due to the reduced pulse fluence (0.2 J/cm^2^) or increased number of scans (9 scans with 10 μm line spacing), or both.

**Figure 6:**
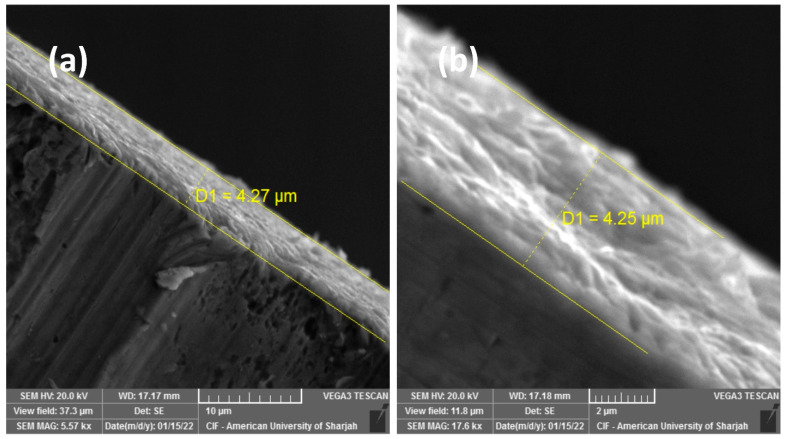
Cross section of the MoO_x_ deposited over mild steel observed at (**a**) 5.57 k× and (**b**) 17.6 k× magnifications.

The topography analysis of the coatings depicts average roughness (R_a_) and root mean square roughness (R_q_) of around 0.27 and 0.33 μm, respectively (Figure S5). Similarly, the thickness of the as-deposited Mo/MS was measured to be around 12 μm (Figure S6a,b), which compressed to 4.25 μm (Figure 6a,b) upon fs laser treatment. The reduction in thickness is indicative of the more compactness of the film. This is because the sequential 1.5 J/cm^2^ and 0.2 J/cm^2^ post-deposition scans fused the ablation debris of the as-deposited Mo/MS sample.

The cross section of MoO_x_/MS was prepared after grinding it with abrasive papers without using any lubricant. The signature of the harsh grinding process is visible on the steel substrate, but we could not identify any huge chunks of MoO_x_ removed during the grinding process, hinting at the amorphous nature of MoO_x_ layer. Despite such a rigid grinding process, the film was found fully intact with the substrate and did not delaminate at all, suggesting its good adhesion with the substrate. We observed the cross section at different locations, but the film was found well intact with the substrate, with no observed delamination. The improved adhesion is most probably due to the Mo–Fe alloy system formation at the interface. The heated Mo in contact with the underlying Fe can easily fuse Fe. As a result, the energetic Fe from the substrate can also marginally diffuse into the top MoO_x_. The strong adhesion of the coat with the substrate suggests that the fs-laser induced instantaneous melting and solidification actually resulted in a strong Mo–Fe bond coat to hold the top MoO_x_ well-adhered to the underlying MS. Though the laser pulse can excite the underlying MS through conduction or convection, it is not probable that the laser penetrates down to directly energize the MS substrate.

The effective penetration depth depends on the material as well as the laser pulse parameters. The ablation threshold of the precursor Mo is around 0.11–0.15 J/cm^2^ [26,56,57,58]. The average penetration depth of Mo was estimated to be around 16.5 nm for 6 fs pulses of fluence 0.15 J/cm^2^ [26]. Similarly, a penetration depth of 6 nm was reported for 500 fs pulses of fluence 0.3 J/cm^2^. The same study reported a penetration depth of around 18 nm for 1.5 J/cm^2^ fluence [56]. Interestingly, low-level ablation off the Mo surface persists even if the fluence was reduced to ¼ the ablation threshold (0.11 J/cm^2^) of Mo [29]. For the photoformed MoO_x_, the average threshold is around 0.6 J/cm^2^ for 8 ps pulses of 1064 nm [59], which is expected to decrease as the pulse duration decreases. This is because longer pulses have lower peak powers [60] and are associated with higher thermal diffusion, and deeper thermal propagation into the sample, therefore requiring relatively more energy than shorter pulses for ablation [59,60,61]. Nonetheless, the ablation threshold is expected to change with each scan due to the laser-induced compositional, morphological, structural, and optical changes associated with each scan [47]. Hence, in our study, it can be inferred that ablation takes place for both the 0.2 J/cm^2^ and 1.5 J/cm^2^ scans; however, for each pulse the ablation rate is of the order of a few nanometers [56]. For 50 μm spot size and 50 kHz repetition rate, a scan speed of 3325 mm/s gave a spot overlap rate of -33%, which is analogous to 16 μm separation between adjacent spots. The 16 μm separation ensured that each pulse hit the same spot only once per line scan. These measures ensured that the laser could not penetrate deep to hit the substrate. The film thickness and morphology are other indicators of the fact that the ablation rate was negligible for the laser to penetrate through the topcoat and hit the substrate. The loosely packed as-deposited 12 μm thick film was reduced to 4.25 μm thick compact film after complete treatment. The uniform coverage of the sample with LIPSS and the absence of ablation craters are further evidence that the applied laser parameters (i.e., pulse duration (40 fs), the pule fluence (0.2–1.5 J/cm^2^), and the scan speed of 3325 mm/s) were optimum for inducing melting without causing excessive ablation.

Figure S2 depicts a mild steel surface structured with the same laser pulse and scan parameters as employed for MoO_x_/MS [56]. If the laser could penetrate into depths of the order of 4.25 μm (which is MoO_x_ thickness) or beyond, features similar to that of Figure S2 would have been observed. The structures for mild steel are due to higher ablation rate experienced at the same laser and scan settings.

**Figure 7:**
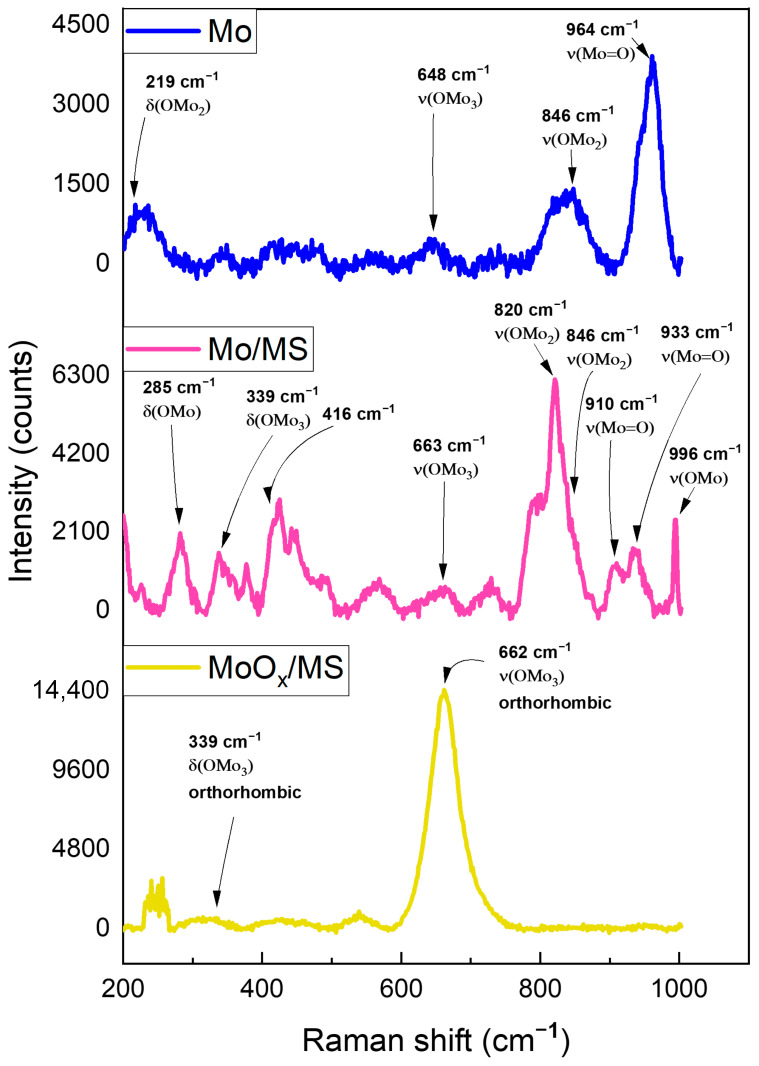
Raman spectra of the molybdenum source (Mo), as-deposited molybdenum (Mo/MS), and fully treated molybdenum to obtain molybdenum oxide (MoO_x_/MS).

We used confocal Raman microscopy to analyze the chemical nature of the sample’s surface. As shown in Figure 7, oxides of Mo were detected along the surface of the precursor Mo sheet, the as-deposited Mo/MS, and the fully treated MoO_x_/MS. We could trace different modes of vibrations in the 200–1000 cm^−1^ range related to different Mo oxide structures. Although Mo oxide exists in many polymorphic forms, the most stable orthorhombic structure of edge-sharing MoO_6_ octahedra was identified from the bands around 996 cm^−1^, 820 cm^−1^, 663 cm^−1^, 339 cm^−1^, and 285 cm^−1^ [62,63,64].

Mo generally prefers to stay in a body-centered cubic (BCC) structure, and it typically crystallizes into a BCC structure upon solidification. In some cases, it deposits as face-centered cubic (FCC) as well [65]. FCC and BCC metals have only acoustic phonons and no optical phonons, which are not detectable with Raman spectroscopy. Therefore, we do not see first-order Mo signals in any Raman spectra [66].

For the fully treated MoO_x_/MS, the preferred vibration mode determined is the three coordinated-edge OMo_3_ stretching and bending vibrations at 662 cm^−1^ and 339 cm^−1^, respectively. These bands signify the presence of a stable orthorhombic structure along the surface [64].

Although MoO_x_/MS was prepared in the air, there was no signature of any significant nitrides content on the surface. This is predominantly due to the reduced laser fluence (i.e., 1.5 J/cm^2^ and 0.2 J/cm^2^) used in the post-treatment process. Since each nitrogen molecule is composed of N atoms bonded by a very strong triple bond that requires 945 kJ mol^−1^ to break it [67], relatively small excitations are inefficient at producing significant quantities of nitrides. In comparison, each oxygen molecule is constituted of oxygen atoms bonded by a double bond between them, and breaking it requires an energy of 498 kJ mol^−1^. Likewise, the ionization potential of nitrogen into N^3−^ equals 47.44 eV and is much higher than that of O to ionize it into O^2−^ that is 35.11 eV. For these reasons, the free energy of formation is less negative for nitrides than that of similar oxides [68]. This is why we can only identify oxides but no nitrides in MoO_x_/MS.

The XRD patterns shown in Figure S7 suggest that there is no long-range order is MoO_x_/MS, though orthorhombic Mo oxide was confirmed from Raman analysis. Similarly, there is no crystalline Mo left in the sample either. The 1.5 J/cm^2^ treated sample is also fully amorphous. The Mo peak could only be observed in Mo/MS at 40.6°, which indicates the preferred (110) texture of cubic Mo [69]. The amorphicity of the post-treated samples is attributed to the short excitation time associated with ultrashort pulses. The localized sudden heating and cooling do not render sufficient time for gradual relaxation. Similarly, O, which attaches to Mo during the laser-scanning process, does not find sufficient time to produce MoO_x_ of large crystallite size.

The resultant film properties are a function of the fluence employed. Likewise, both physical and chemical properties of the film are subject to change with each scan. The number of successive scans play an essential role in shaping these properties. Since the sample was scanned at two different fluence values, i.e., at 1.5 J/cm^2^, followed by 8 times scanning at 0.2 J/cm^2^, different intrinsic film properties are anticipated for the two fluence regimes.

Among the fluence-dependent properties are chemical, structural, optical, and morphological characteristics of the topcoat. Due to the higher accumulated fluence, the fully treated i.e., 1.5 J/cm^2^ followed by 0.2 J/cm^2^ scanned sample (MoO_x_/MS) is more oxidized than the1.5 J/cm^2^ scanned sample (Mo-1.5/MS). This is due to the laser-induced oxygen plasma reacting with the topcoat and oxidizing it. Oxygen diffusion due to thermal and concentration gradients as well as melt hydrodynamic movement are associated phenomena for enhanced oxidation at higher accumulated fluence [70]. The O content in the interior of the MoO_x_ layer may not be the same as that at the exterior surface. Actually, the O content may decrease as we traverse down towards the substrate. The microstructure and crystallinity are also affected by the instantaneous melting and solidification due to ultrafast laser-matter interaction. Usually amorphous/nanocrystalline films are obtained after short laser–matter interaction. The crystallinity has been evidenced to improve with increasing accumulated fluence [70]. This is because higher accumulated fluence induces more reactive oxygen species [71], which not only enhance the MoO_x_ content but also improve the crystallinity of the topcoat [72,73]. Similarly, the high transparency of MoO_x_/MS relative to Mo-1.5/MS is due to the enhanced MoO_x_ content that improved the overall transparency of MoO_x_/MS [74]. Among the morphological transformations, the most appealing morphological change was the appearance of HSFL and quasi-periodic grooves along with LSFL on the fully treated MoO_x_/MS. Changes in the ablation threshold of the topcoat due to compositional, morphological, structural, and optical changes associated with each scan are also inevitable [47].

As mentioned previously, although Mo and its compounds are used for many technological applications, we are more interested in determining the quality of the coating. As an application example, MoO_x_/MS was tested for corrosion inhibition in a simulated 3.5 wt% aqueous NaCl environment. The performance was evaluated in terms of corrosion inhibition efficiency (CIE), which was, in turn calculated using the relation (1) given below.

(1) $$\mathrm{CIE}=\frac{J_{\mathrm{cor}\;\left(\mathrm{MS}\right)}-J_{\mathrm{cor}\;\left(\mathrm{suppresant}\right)}}{J_{\mathrm{cor}\;\left(\mathrm{MS}\right)}}$$

**Figure 8:**
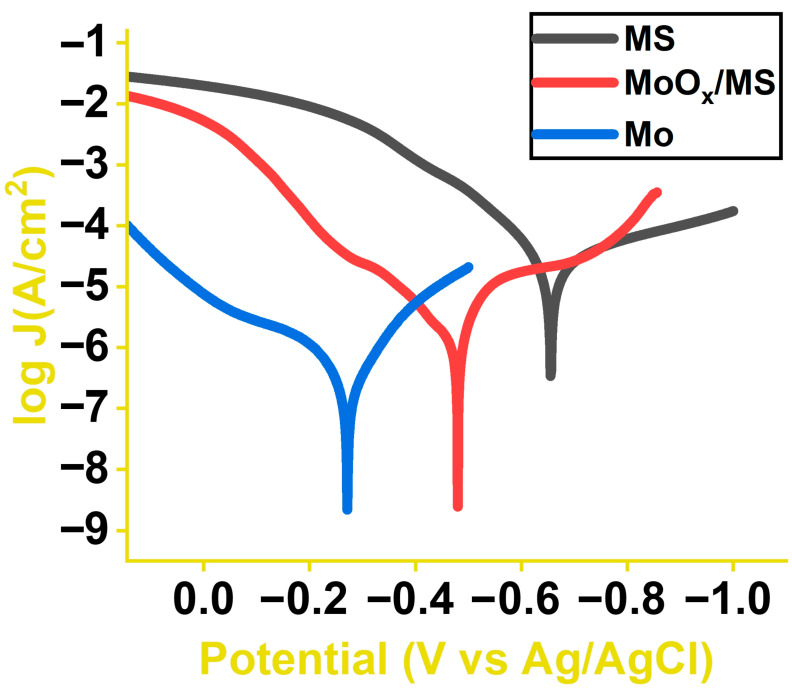
Tafel plots of mild steel, molybdenum oxide deposited over mild steel, and molybdenum foil obtained in 3.5 wt% aqueous NaCl solution.

Here, $J_{\mathrm{cor}\;\left(\mathrm{MS}\right)}$ and $J_{\mathrm{cor}\;\left(\mathrm{suppresant}\right)}$ represent the corrosion current densities measured for the polished and MoO_x_ coated mild steel, respectively. The average CIE improvement recorded relative to polished MS was 94.83% (Figure 8 and Table 1). The average value was calculated by testing and averaging 5 MoO_x_/MS samples. The precursor pristine Mo had an average CIE of 98.65% in an aqueous NaCl environment. Since MoO_x_/MS is substoichiometric and the oxygen content is less saturated, any oxygen vacancy will leave behind a Mo-dangling bond [75]. These dangling bonds and the higher surface area due to the laser-induced surface structures are believed to be the reasons for the lower CIE relative to pristine Mo. The CIE of MoO_x_/MS samples could be further improved by decreasing the surface area and fully oxygenating the sample.

## 4. Conclusions

A thin film deposition technology solely based on fs laser was introduced for depositing amorphous coatings of relatively high melting point materials (in this case, Mo). Despite the very high deposition rates achievable with this technique, the films are uniform, crack-free, pore-free, and well-adhering to the substrate. The method can be employed to achieve superb adhesion without substrate heating or roughening and with no bond coats required. The method comprises a very raw coating of the source material being deposited in a vacuum chamber employing a high-rate femtosecond pulsed laser deposition (fs-PLD), followed by thoroughly scanning the raw sample to transform it into a high-quality coating. As a manifestation of the technique, a raw Mo film of around 12 μm thickness was deposited from a rotating molybdenum source in a vacuum chamber at 0.5 J/cm^2^. To obtain a good quality substoichiometric molybdenum oxide layer from the raw deposited Mo, it was further scanned thoroughly, first with 1.5 J/cm^2^ and then with 0.2 J/cm^2^ pulse fluence, while maintaining a scan rate and line spacing of 3325 mm/s and 10 μm, respectively. The substoichiometric molybdenum oxide’s corrosion inhibition efficiency (CIE) was analyzed in a 3.5 wt% aqueous NaCl environment. Compared to polished mild steel, an average corrosion inhibition efficiency of 94.83% was achieved.

## Figures and Tables

**Table 1:** Polarization resistance and corrosion current calculated from Tafel extrapolation and compared.

Nomenclature	$\mathrm{Linear}\;\mathrm{Polarization}\;\mathrm{Resistance},\;R_{pol}$ (Ω)	$\mathrm{Corrosion}\;\mathrm{Current}\;\mathrm{Density},\;J_{cor}$ $\left(\mu A/cm^{2}\right)$	Corrosion Inhibition Efficiency, CIE(%)
Mild steel (MS)	1312	30.510	
MoOx/MS	10,953	1.567	94.83 ± 1.67%
Mo foil	80,445	0.412	98.65 ± 0.88%

## Data Availability

Data underlying the results presented in this paper are not publicly available at this time but may be obtained from the authors upon reasonable request.

## Supplementary Materials

The following supporting information can be downloaded at: https://www.mdpi.com/article/10.3390/ma16030909/s1.
