# Supplementary material for: A Two-Step Femtosecond Laser-Based Deposition of Robust Corrosion-Resistant Molybdenum Oxide Coating
Source: Materials (Basel). 2023 Jan 18;16(3):909. doi: 10.3390/ma16030909 (PMC9918068; doi:10.3390/ma16030909)
Supplement: Supplementary file 1 [file materials-16-00909-s001.zip › materials-2036473-supplementary.pdf]

Supplementary Information

A two-step femtosecond laser-based deposition of robust corrosion-resistant molybdenum oxide coatings

Asghar Ali[a,b], Piotr A. Piatkowski[b], Tahir Nawaz[b], Shahbaz Ahmad[b], Taleb Ibrahim[b,c], Mustafa Khamis[b,d], and Ali S. Alnaser[a, b,*]

[a]Department of Physics, American University of Sharjah, Sharjah 26666, United Arab Emirates

[b]Materials Science and Engineering Program, College of Arts and Sciences, American University of Sharjah, Sharjah 26666, United Arab Emirates

[c]Department of Chemical Engineering, College of Engineering, American University of Sharjah, Sharjah 26666, United Arab Emirates

[d]Department of Biology, Chemistry, and Environmental Sciences, College of Arts and Sciences, American University of Sharjah, Sharjah 26666, United Arab Emirates

*Email: aalnaser@aus.edu

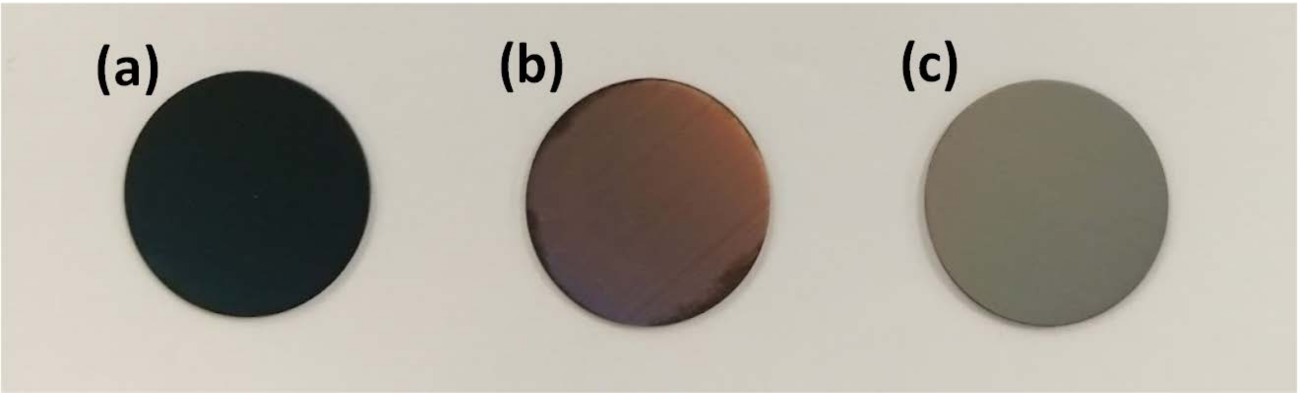

Figure S1. Camera captured images of the (a) as-deposited Mo over mild steel obtained via fs-PLD at 0.5 J/cm^2 fluence, 240 fs pulse duration, and 150 kHz repetition rate (Mo/MS) (b) sample obtained after scanning Mo/MS with 40 fs and 50 kHz pulses of 1.5 J/cm^2 fluence (Mo-1.5/MS) (c) sample obtained after scanning Mo-1.5/MS surface with 40 fs and 50 kHz pulses of fluence 0.2 J/cm^2

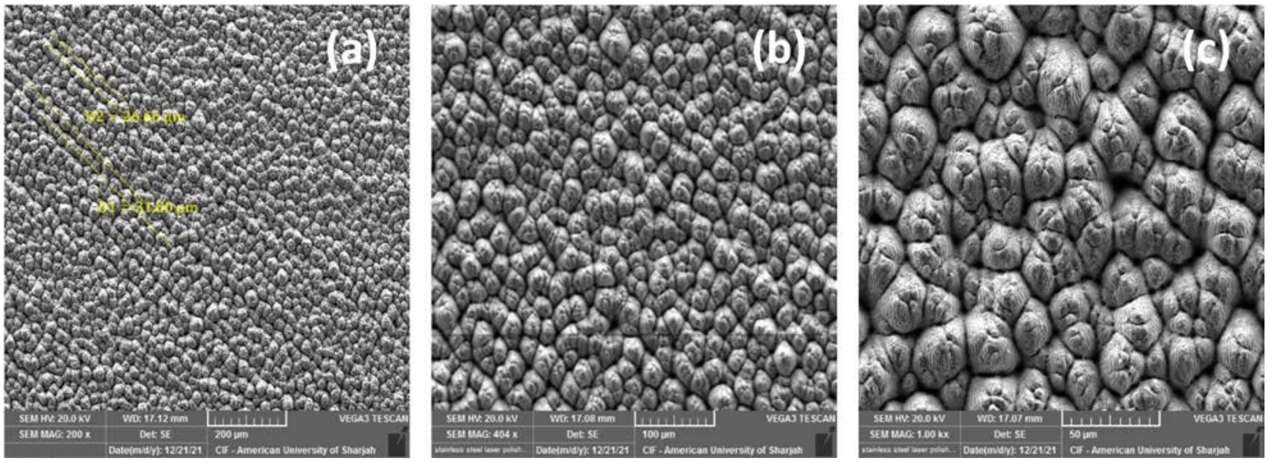

Figure S2. Morphology at (a) 200 x, (b) 404 x, and (c) 1 kx after of mild steel after laser-scanned with 40 fs and 50 kHz pulses of fluence 1.5 J/cm^2, followed by 0.2 J/cm^2

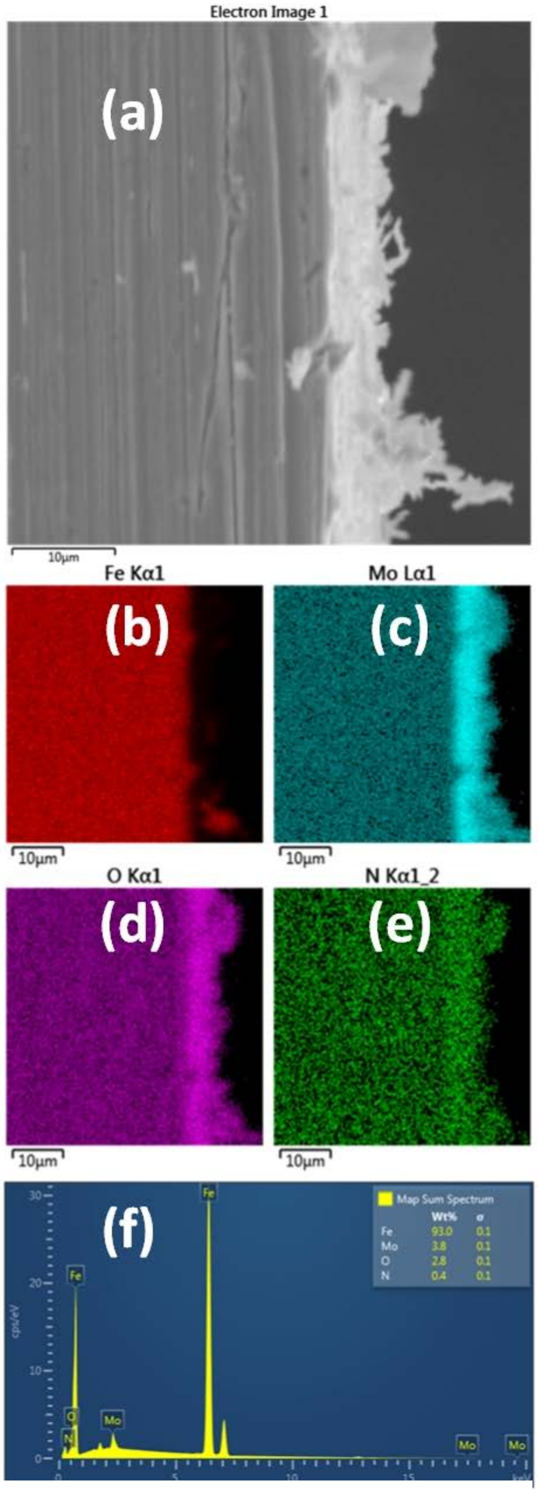

Figure S3. EDS analysis of the cross section of the as deposited Mo over mild steel

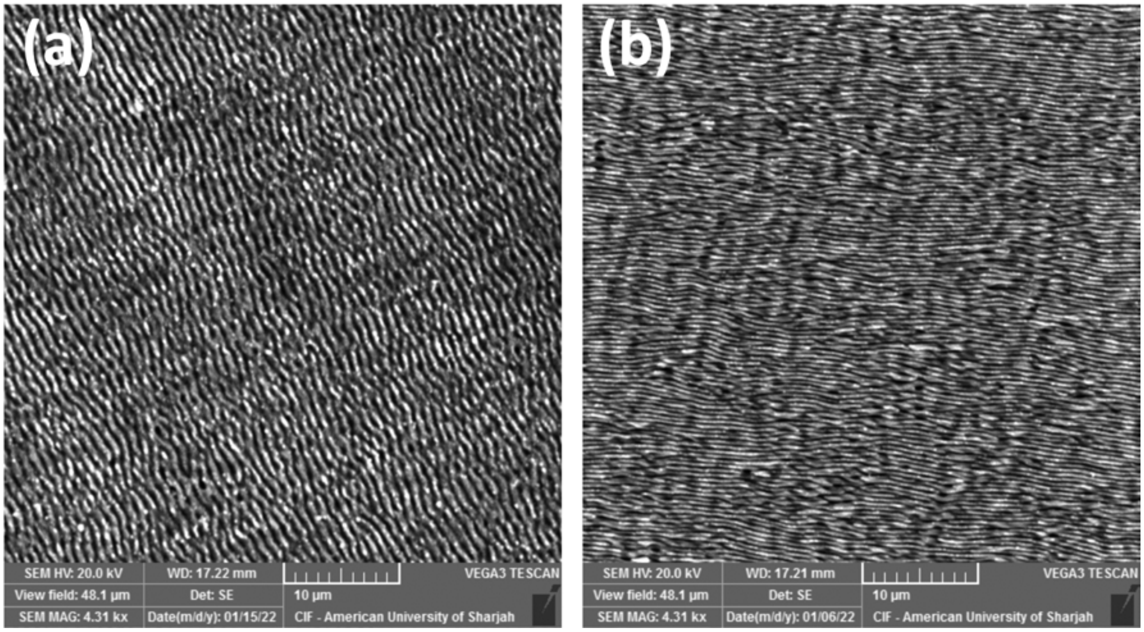

Figure S4. SEM micrographs of (a) Mo-1.5/MS and (b) MoO_x/MS with a view field of 48.1 μm for 2D FFT analysis

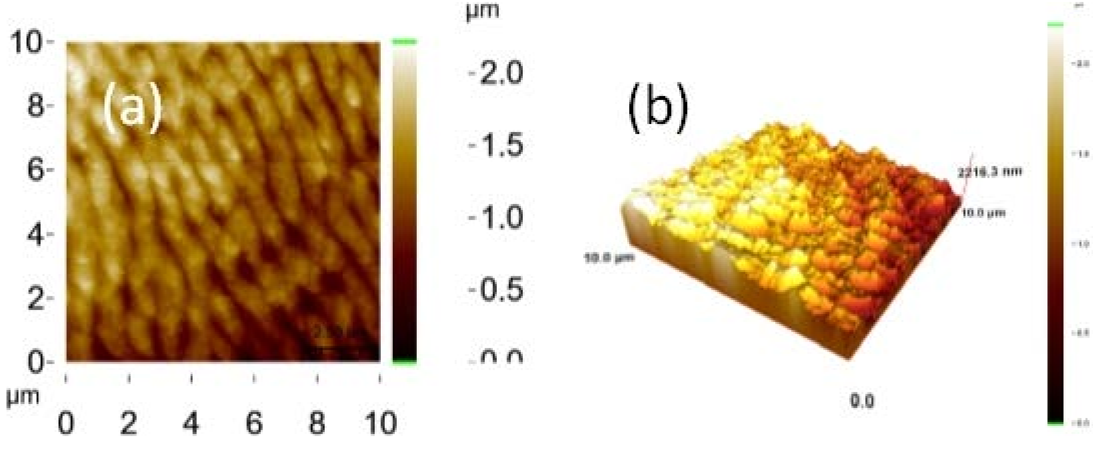

Figure S5. AFM topography of Mo/MS (a) top view (b) 3D profile

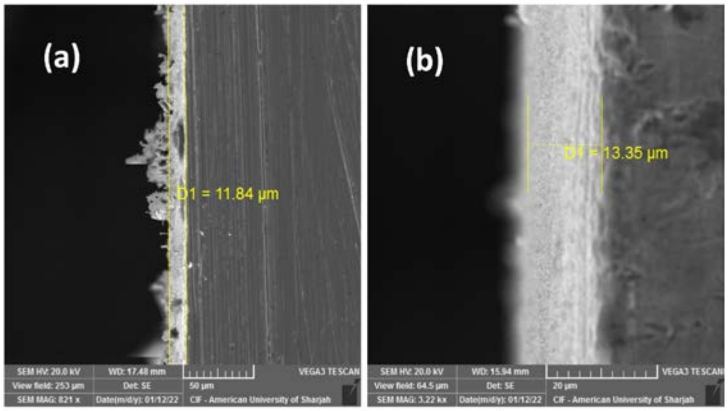

Figure S6. Cross sectional SEM micrograph of the as-deposited Mo/MS acquired at (a) 821 x and (b) 3.22 kx magnifications

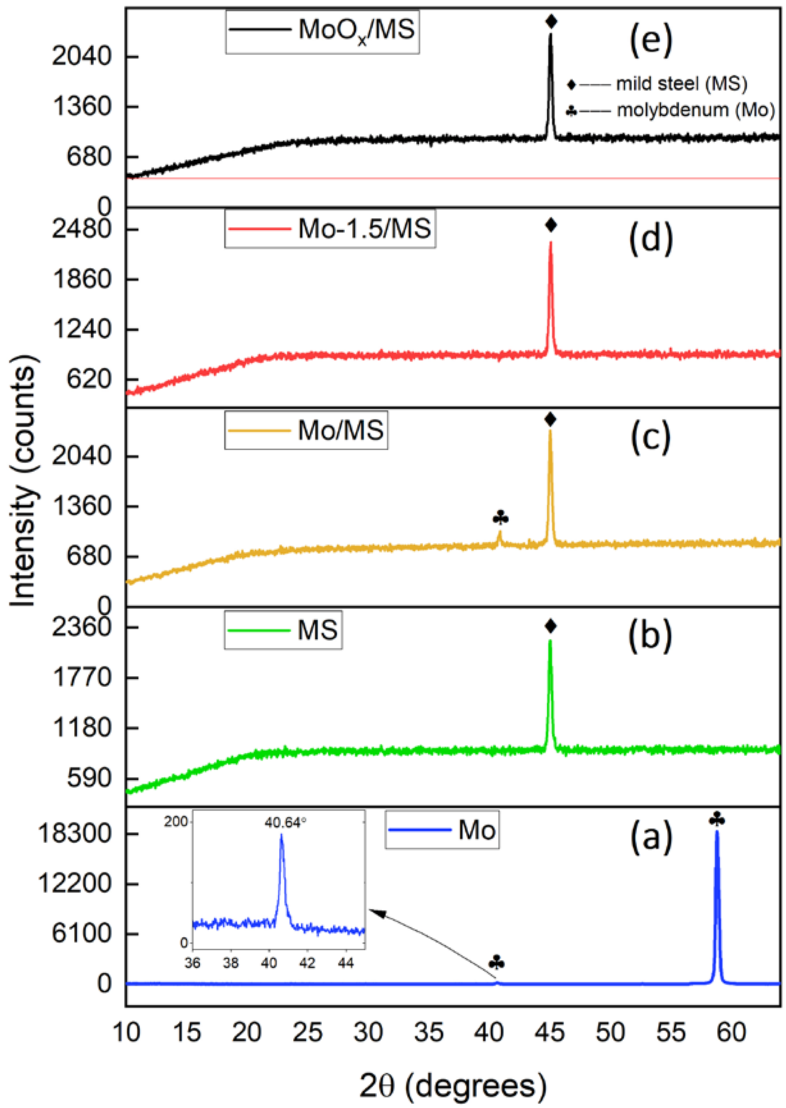

Figure S7. X-ray diffraction analysis of **(a)** the source molybdenum foil (Mo), **(b)** mild steel (MS), **(c)** as deposited molybdenum over mild steel (Mo/MS), and molybdenum oxide coating over mild steel after laser-scanning Mo/MS with fluence of **(d)** 1.5 J/cm^2, followed by **(e)** 0.2 J/cm^2

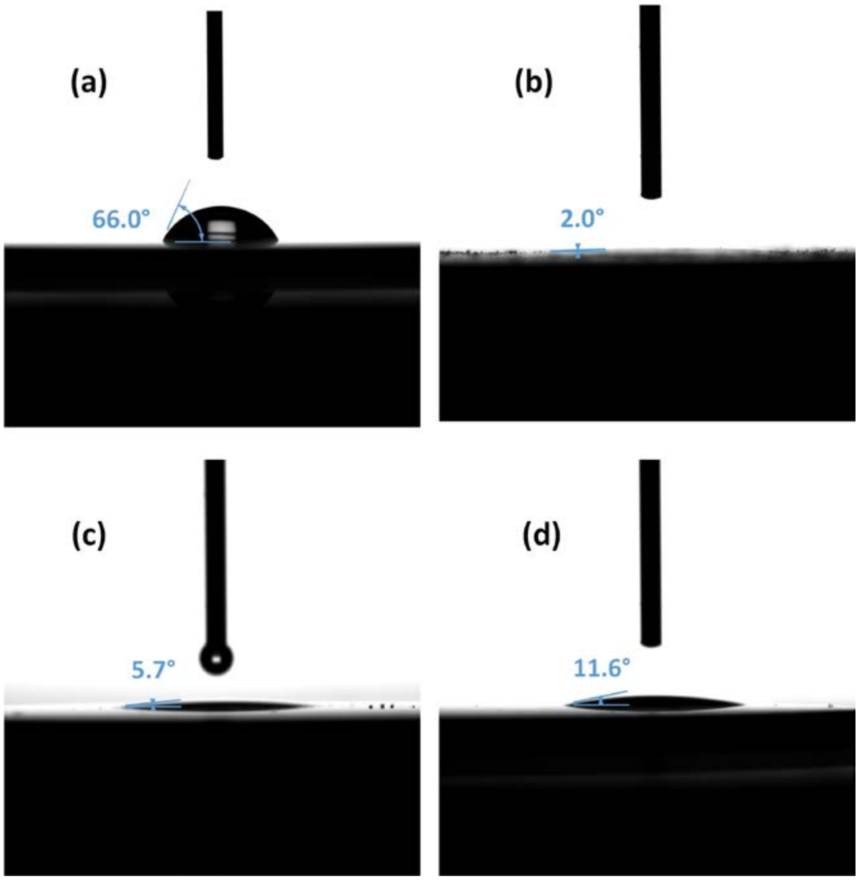

Figure S8. Contact angle measurements of (a) polished mild steel (MS), (b) as deposited Mo (Mo/MS), (c) 1.5 J/cm^2 laser-scanned (Mo-1.5/MS) and (d) 0.2 J/cm^2 laser-scanned after 1.5 J/cm^2 treatment in air (MoO_x/MS)
